# Transactions of the Medical Society of the State of New York for 1859

**Published:** 1859-06

**Authors:** 


*Transactions of the Medical Society of the State of New York. For the year* 1859. Albany, Charles Van Benthuysen, printer. 1859.

We have received, through the kindness of Prof. Hamilton, a copy of the New York State Society's Transactions. This we will take occasion to notice more fully hereafter. For the present we will only call attention, by his request, to a couple of serious errors of the printer, contained in his paper on "Prognosis in Fractures of the Neck of the Femur within the Capsule." Malgaigne is constantly spelt Malgaique, and Swan is constantly spelt Severn ("Severn's case," pp. 34, 35).

